# In patients with lung cancer is combined endobronchial ultrasound and endoscopic ultrasound superior to conventional mediastinoscopy in staging the mediastinum?

**DOI:** 10.1016/j.amsu.2021.102953

**Published:** 2021-10-14

**Authors:** Aaron Gunawan, Lucy Manuel, Laura S. Fong, Levi Bassin

**Affiliations:** a St Vincent's Hospital Clinical School, University of New South Wales, Randwick, NSW, Australia; b Department of Cardiothoracic Surgery, Royal North Shore Hospital, St Leonards, NSW, Australia; c Department of Cardiothoracic Surgery, Liverpool Hospital, Liverpool, NSW, Australia

**Keywords:** endobronchial Ultrasonography, Endoscopic ultrasonography, Mediastinoscopy, Lung cancer, Staging

## Abstract

A best evidence topic in thoracic surgery was written according to a structured protocol. The question addressed was ‘In patients with lung cancer, is combined endobronchial ultrasound and endoscopic ultrasound (EBUS + EUS) superior to cervical mediastinoscopy (CM) in staging the mediastinum?’ Altogether more than 110 papers were found, of which one meta-analysis, two RCTs, and two cohort studies represented the best evidence to answer the clinical question. The authors, journal, date and country of publication, patient group studied, study type, relevant outcomes and results of these papers are tabulated. Studies directly comparing EBUS + EUS and CM are limited in number and quality, with the majority of studies focusing on comparing endosonographic techniques or a single technique with surgical staging. Moreover, in four out of five studies, surgical staging of the mediastinum was undertaken following a negative EBUS + EUS result, limiting the utility of comparing endosonography alone. Regardless of this, the initial EBUS + EUS approach followed by surgical staging if negative resulted in greater sensitivity and detection of N2/3 metastases as well as greater sampling in the majority of studies, resulting in higher likelihood of upstaging and treatment alterations for patients. There was also improved quality of life demonstrated in the EBUS + EUS group with significant reductions in futile thoracotomies and less complications when compared with exclusive CM staging. We conclude that a combined approach of combined endosonography in the first instance, followed by CM staging of the mediastinum results in greater sensitivity of nodal disease and subsequent greater accuracy in upstaging and determining treatment plans with a concurrent reduction in complication rates and futile procedures.

## Highlights

• Mediastinal assessment is essential for management and for lung cancer patients.
• Controversy remains regarding the optimal method.
• CM remains gold standard, however, EBUS + EUS has been shown to be equivalent.
• Greater sensitivity is achieved with a combined endosongraphy and surgical approach.

## 1 Introduction

A best evidence topic was constructed according to a structured protocol. This is fully described in the IJS [1].

## 2 Clinical scenario

You attend a lung cancer multidisciplinary meeting involving respiratory physicians, oncologists and thoracic surgeons where they are discussing a 62-year-old man with a 45 mm right upper lobe non-small cell lung carcinoma. Positron-Emission Tomography (PET) reveals low grade uptake of flurodeoxyglucose (FDG) at stations 4R and 7. A colleague suggests that combined endobronchial ultrasound (EBUS) and endoscopic ultrasound (EUS) would be less invasive and more accurate than proceeding directly to cervical mediastinoscopy (CM), the gold standard staging procedure. You resolve to review the scientific literature to inform your plan.

## 3 Three-part question

In [patients with lung cancer], is [combined endosonography (EBUS + EUS)] superior to [Cervical Mediastinoscopy] in staging the mediastinum?

## 4 Search strategy

A literature search was performed on MEDLINE database (from 1946 to present) using the OVID interface with the terms: (EBUS OR endobronchial) AND (EUS OR endoscopic) AND (combined OR endosonography OR ((ultrasonography) OR (ultrasound))) AND mediastinoscopy AND staging AND (lung neoplasms OR lung cancer). Only papers published in English were reviewed. The reference lists of initially identified papers were searched for relevant studies.

## 5 Search outcome

A total of 110 papers were found using the reported search. From these, five papers were identified that were deemed to provide the best evidence to answer the question. These are presented in Table 1.

**Table 1:** Best evidence papers.

Author, date, journal and country, study type (level of evidence)	Patient group	Outcomes	Key results EBUS + EUS vs. CM	Comments
[2] Annema et al., 2010, JAMA, Netherlands RCT (level II)	241 patients EBUS + EUS: 123 CM: 118	Detection of nodal metastases	46% v. 35% (p = 0.11)	Greater sensitivity (94%) by combining endosongraphy followed by surgical staging (p = 0.02) Of the endosongraphy group, 65 patients went on to have additional surgical staging- which detected nodal metastases in 6 additional patients (9%)
		Sensitivity (without additional surgical staging)	85% vs. 79% (p = 0.47)	
		NPV (without additional surgical staging)	85% vs. 86% (p= >0.99)	
		Detection of N2/N3 metastases	50% vs. 35% (**p** = **0.02)**	
		Detection of locally advanced/invasive disease	52% vs. 36% **(p** = **0.01)**	
		Unnecessary thoracotomy	7% vs. 18% **(p** = **0.02)**	
		Complications (without additional surgical staging)	6% vs. 1% **(p** = **0.03)**	
[3] Liberman et al., 2014, Chest, Canada Prospective Cohort Study (level IIa)	166 patients EBUS + EUS: 166 CM:166 Both procedures undertaken, patient acts as own control	Mean number of lymph node stations sampled	3.9 vs. 3.1	Sensitivity EBUS + EUS = 91% 5 patients where EBUS + EUS was negative and CM revealed N2 disease Endosonography led to diagnosis N2/N3/M1 disease in 24 patients where CM findings were negative, preventing futile thoracotomy in 14% of patients
		Prevalence of N2/N3 disease	53/166 (32%)	
		NPV	92% vs. 89% (p = NR)	
		Diagnostic accuracy	91% vs. 89% (p = NR)	
		Complications	0 vs. 15 (p = NR)	
[4] Sehgal et al., 2016, Ann Thorac Surg, India Meta-analysis (level Ib)	9 studies between 2005 and 2015 including 960 patients	Pooled RD of diagnostic sensitivity	0.11 (CI 0.07–0.29)	3 studies: EUS vs. CM 4 studies: EBUS vs. CM 2 studies: EUS + EBUS vs. CM Similar outcomes in stations 7 and 4R
		Pooled RD of complication rate	6.8% (CM higher; CI 4.3–9.8)	
		Sensitivity analysis lymph node station 2R	RD 0.16 (EBUS + EUS higher; **p** = **0.003**)	
		Sensitivity analysis lymph node station 4L	RD 0.19 (EBUS + EUS higher; **p** = **0.0002**)	
[5] Zielinski et al., 2013 J Thorac Oncol, Poland Retrospective Cohort Study (level III)	623 patients EBUS: 351 EUS: 72 CUS: 200 TEMLA: 276	Primary staging sensitivity	87.8% vs. 96.2% (**p** < **0.01**)	TEMLA only performed following negative EBUS/EUA- positive in 50 patients (43 N2, 7 N3)
		Primary staging specificity	98.7 vs. 100% (**p** = **0.03**)	
		Primary staging NPV	82.5% vs. 99.6% (**p** < **0.01**)	
		Primary staging PPV	99.1% vs. 100% (p = 0.07)	
		Primary staging prevalence	63.1% vs. 18.4% (**p** < **0.01**)	
		Re-staging sensitivity	64.3% vs. 100% (**p** < **0.01**)	
		Re-staging specificity	100% vs. 100% (p = 1.00)	
		Re-staging NPV	82.1% vs 100% (**p** < **0.01**)	
		Re-staging PPV	100% vs. 100% (p = 1.00)	
		Re-staging prevalence	40% vs. 19.2% (P **< 0.01**)	
		Mean number of nodes sampled	3.7 vs. 32.8	
		Morbidity	0 vs. 7.2% (p = NR)	
[6] Sharples et al., 2012, Health Technology Assessment, UK RCT (level II)	241 patients EBUS + EUS: 123 CM: 118	Sensitivity for detecting N2/3 metastases (combined EBUS + EUS + CM if negative vs. CM)	94% vs. 79% (**p** = **0.02**)	Patients proceeded to surgical staging if EBUS + EUS is negative Surgical staging was avoided due to endosonography findings in 47% of patients
		NPV (combined EBUS + EUS + CM if negative vs. CM)	93% vs. 86% (p = 0.18)	
		Sensitivity for detecting N2/3 metastases (combined EBUS + EUS only vs. CM)	85% vs. 79% (p = 0.62)	
		NPV (combined EBUS + EUS only vs. CM)	85% vs. 86% (p = 1.00)	
		Unnecessary thoracotomy	7% vs. 18% (**p** = **0.02**)	
		Complications	5% vs. 6% (p = 0.78)	
		Survival over 6 months	9 vs. 11 deaths (p = 0.57)	
		European quality of life end of staging	0.117 in favour of EBUS + EUS (**p** = **0.003**)	

EBUS- Endobronchial Ultrasound; EUS- Endoscopic Ultrasound; CM- Mediastinoscopy; CUS- Combined EBUS + EUS; TEMLA- Transcervical Extended Mediastinal Lymphadenectomy; PPV- Positive Predictive Value; NPV- Negative Predictive Value; RD- Risk Difference.

## 6 Results

The results of the five papers (one meta-analysis, two RCTs, and two cohort studies) are summarised in Table 1.

## 7 Discussion

Annema et al. [2] performed a multi-centre RCT involving 241 patients comparing EBUS + EUS with CM which demonstrated no significant difference in sensitivity at detecting mediastinal nodal disease (85% vs. 79%, p = 0.47). However, EBUS + EUS followed by CM was superior to CM alone, with statistically greater sensitivity (94% vs 79%, p = 0.02). Additionally, EBUS + EUS was superior in detecting N2/3 nodal disease, as well as locally advanced and invasive disease (50% vs. 35%, p = 0.02; 52% vs. 36%, p = 0.01). There were significantly more unexpected stage IV disease found at the time of thoracotomy for planned lung resection in the CM group, as well as more complications (7% vs. 18%, p = 0.02; 6% vs. 1%, p = 0.03).

Liberman et al. [3] conducted a single-centre prospective study in 166 patients comparing EBUS + EUC with CM. All patients in the study had EBUS + EUS first followed by CM - acting as a control for themselves. The sensitivity of EBUS + EUS was 91% with a further 5 patients diagnosed with N2 disease following CM. EBUS + EUS found an additional 24 patients (14.4%) with N2/N3 disease over CM, which may have prevented futile thoracotomies in a significant proportion of patients. The negative predictive value and diagnostic accuracy were similar among the techniques (92% vs. 89%, p = NR; 91% vs. 89%, p = NR), with more complications occurring during the CM procedure (0 vs. 15, p = NR). Unfortunately, statistical analysis was not performed directly comparing the two techniques, thus limiting the utility of this study.

Sehgal et al. [4] performed a meta-analysis including 9 studies between 2005 and 2015 that included 960 patients. The authors demonstrated equivalence between EBUS + EUS and CM with a pooled risk difference (RD) for diagnostic sensitivity of 0.11 (CI 0.07–0.29) across all studies. Additionally, endosonography had a higher pooled RD in terms of complications with a 6.8% increase compared with CM (CI 4.3–9.8). Significantly greater sensitivities were reported for lymph node stations 2R and 4L (RD 0.16, p = 0.03; RD 0.19, p = 0.002), however, there were similar outcomes in stations 7 and 4R.

Zielinski et al. [5] completed a retrospective cohort study involving 623 patients who had endosonography or surgical Transcervical Extended Mediastinal Lymphadenectomy (TEMLA) primary staging for non-small cell lung cancer. Surgical staging demonstrated greater sensitivity (87.8% vs. 96.2%, p < 0.01), specificity (98.7% vs. 100%, p = 0.03) and negative predictive value (82.5% vs. 99.6%, p < 0.01). TEMLA was only performed following a negative EBUS + EUS result, which detected additional N2/N3 disease in 50 patients. Separate analysis was also completed for re-staging procedures, where TEMLA also had significantly higher sensitivity (64.3% vs. 100%, p < 0.01) and negative predictive value (82.1% vs. 100%, p < 0.01). There was higher morbidity and mortality in the surgical group, however, analysis was not performed to evaluate significance (0 vs. 7.2%, p = NR).

Sharples et al. [6] completed an RCT with 241 patients involving four districts in the UK between 2007 and 2009. Patients were randomised to undergo EBUS + EUS or CM alone, with patients proceeding to CM if EBUS + EUS was negative. This RCT demonstrated that using combined EBUS + EUS, followed by CM if negative, had a higher sensitivity and negative predictive value than CM alone (94% vs. 79%, p = 0.02; 93% vs. 86%, p = 0.18). Conversely, when the diagnostic accuracy of EBUS + EUS alone is assessed, without additional CM, the results were not statistically significant (sensitivity 85% vs. 79%, p = 0.62; NPV 85% vs. 86%, p = 1.00). CM was avoided in 47% of patients due to positive endosonography findings. Additionally, unnecessary thoracotomy was higher in the CM staging group (7% vs. 18%, p = 0.02), however, complications were similar between the groups (5% vs. 6%, p = 0.78).

There was no difference in survival at 6 months for EBUS + EUS compared with CM (9 vs. 11 deaths, p = 0.57). Quality of life was significantly improved in the EBUS/EUS cohort as demonstrated by a higher European quality of life staging score (EQ-5D 0.117, p = 0.003).

## 8 Clinical bottom line

Studies directly comparing EBUS + EUS and CM are limited in number and quality, with the majority of studies focusing on comparing endosonographic techniques or a single technique with cervical mediastinoscopy. Moreover, in four out of five studies CM was undertaken following a negative EBUS + EUS result, limiting the utility of comparing endosonography alone. Regardless of this, the initial EBUS + EUS approach followed by surgical staging if negative resulted in greater sensitivity and detection of N2/3 metastases as well as greater sampling in the majority of studies, resulting in higher likelihood of upstaging and treatment alterations for patients. There was also improved quality of life demonstrated in the endosongraphy group with significant reductions in futile thoracotomies and less complications when compared with exclusive CM surgical staging. We conclude that a combined approach of endosongraphy (EBUS + EUS) in the first instance, followed by surgical staging of the mediastinum with CM results in greater sensitivity of nodal disease and subsequent greater accuracy in upstaging and determining treatment plans with a concurrent reduction in complication rates and futile procedures.

## Provenance and peer review

Not commissioned, externally peer reviewed.

## Declaration of competing interest

None declared.
