# Supplementary material for: In patients with lung cancer is combined endobronchial ultrasound and endoscopic ultrasound superior to conventional mediastinoscopy in staging the mediastinum?
Source: Ann Med Surg (Lond). 2021 Oct 14;71:102953. doi: 10.1016/j.amsu.2021.102953 (PMC8531538; doi:10.1016/j.amsu.2021.102953)
Supplement: Multimedia component 1 [file mmc1.docx]

**Annals of Medicine and Surgery**

The following information is required for submission. Please note that failure to respond to these questions/statements will mean your submission will be returned. If you have nothing to declare in any of these categories then this should be stated.

**Please state any conflicts of interest**

All authors must disclose any financial and personal relationships with other people or organisations that could inappropriately influence (bias) their work. Examples of potential conflicts of interest include employment, consultancies, stock ownership, honoraria, paid expert testimony, patent applications/registrations, and grants or other funding.

| Nil conflict of interest to declare |
| --- |

**Please state any sources of funding for your research**

All sources of funding should be declared as an acknowledgement at the end of the text. Authors should declare the role of study sponsors, if any, in the collection, analysis and interpretation of data; in the writing of the manuscript; and in the decision to submit the manuscript for publication. If the study sponsors had no such involvement, the authors should so state.

| Nil funding for research |
| --- |

**Ethical Approval**

Research studies involving patients require ethical approval. Please state whether approval has been given, name the relevant ethics committee and the state the reference number for their judgement.

| Nil ethics approval required |
| --- |

**Consent**

Studies on patients or volunteers require ethics committee approval and fully informed written consent which should be documented in the paper.

Authors must obtain written and signed consent to publish a case report from the patient (or, where applicable, the patient's guardian or next of kin) prior to submission. We ask Authors to confirm as part of the submission process that such consent has been obtained, and the manuscript must include a statement to this effect in a consent section at the end of the manuscript, as follows: "Written informed consent was obtained from the patient for publication of this case report and accompanying images. A copy of the written consent is available for review by the Editor-in-Chief of this journal on request”.

Patients have a right to privacy.  Patients’ and volunteers' names, initials, or hospital numbers should not be used.  Images of patients or volunteers should not be used unless the information is essential for scientific purposes and explicit permission has been given as part of the consent.  If such consent is made subject to any conditions, **the Editor in Chief** must be made aware of all such conditions.

Even where consent has been given, identifying details should be omitted if they are not essential.   If identifying characteristics are altered to protect anonymity, such as in genetic pedigrees, authors should provide assurance that alterations do not distort scientific meaning and editors should so note.

| N/A- nil patients/volunteers |
| --- |

**Author contribution**

Please specify the contribution of each author to the paper, e.g. study concept or design, data collection, data analysis or interpretation, writing the paper, others, who have contributed in other ways should be listed as contributors.

| Aaron Gunawan- Literature search, analysis and writing of manuscript  Lucy Manuel- Literature search, analysis and writing of manuscript  Laura Fong- Analysis and editing of manuscript  Levi Bassin- Analysis and editing of manuscript |
| --- |

**Registration of Research Studies**

In accordance with the Declaration of Helsinki 2013, all research involving human participants has to be registered in a publicly accessible database.  Please enter the name of the registry and the unique identifying number (UIN) of your study.

You can register any type of research at <http://www.researchregistry.com> to obtain your UIN if you have not already registered. This is mandatory for human studies only.  Trials and certain observational research can also be registered elsewhere such as: [ClinicalTrials.gov](http://ClinicalTrials.gov) or ISRCTN or numerous other registries.

| 1. Name of the registry: NA- nil human particpants 2. Unique Identifying number or registration ID: 3. Hyperlink to your specific registration (must be publicly accessible and will be checked): |
| --- |

**Guarantor**

The Guarantor is the one or more people who accept full responsibility for the work and/or the conduct of the study, had access to the data, and controlled the decision to publish

| Levi Bassin |
| --- |
